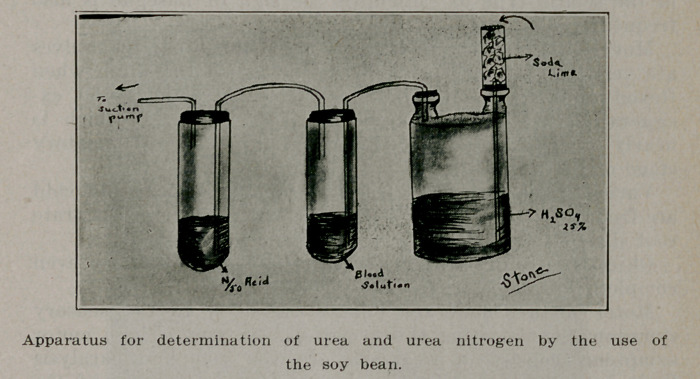# Blood and Urine, Nitrogen and Urea

**Published:** 1916-08

**Authors:** 


### ABSTRACTS.

Have you ever felt that this department of the BUFFALO MEDICAL JOURNAL was lacking in abstracts of your specialty, or that such as were published lacked the expert judgement which you yourself could have exercised in selection and preparation? If not, you are more charitable than the editor has been to himself. If so, will you assist in abstracting from a few journals, and thus make this department more representative and more valuable? Incidentally, there are few forms of study more helpful to the worker than this.

**Blood and Urine, Nitrogen and Urea.** Chester T. Stone, Brooklyn, Med. Times, Mch. (Cut by courtesy of author and editor).

An easy and efficient method of determining urea and urea nitrogen has been solved by the use of the soy bean. The apparatus may or may not have the Wolf bottle as shown. In case it is not used the drying tube is attached to the blood solution tube.

Apparatus for determination of urea and urea nitrogen by the use of the soy bean.

**Urine.**—Take 5 c.c. urine diluted ten times, add one c.c. amyl alcohol, one c.c. 15% soy bean* close and let stand 15 minutes to 20° C, or 3 minutes at 50° C. The tube marked N/50 Acid contains 25 c.c. 50th normal HC1. Areate for one half minute after reaction is complete, quickly open tube and add dry potassium carbonate 4-5 grams, close and areate 15 minutes when all the ammonia gas will have been carried over into the acid. Titrate the acid solution to neutrality with N/50 NaOH. indicator one drop 1% sodium alizarin sulphonate solution. The number of c.c. of N/50 acid neutralized by the ammonia is multiplied by .12 to give the per cent. urea, or by .056 to give per cent. urea nitrogen.

**Blood.**—Take 5 c.c. fresh drawn blood in one c.c. 5% citrate solution and add a few drops amyl alcohol and one c.c. 10% solution soy bean. Proceed as for urine except 10 c.c. of acid are used and .012 is multiplied for per cent. urea or .0056 for per cent. urea nitrogen.

## Figures and Tables

**Figure f1:**